# Effects of a Noncontact Visit Program in the NICU for the Prevention of COVID-19

**DOI:** 10.3390/healthcare11152152

**Published:** 2023-07-28

**Authors:** Hye Young Ahn, Hee Jee Jo, Hyun Jeong Ko

**Affiliations:** College of Nursing, Eulji University, Uijeongbu 11759, Republic of Korea

**Keywords:** high-risk neonate, high-risk neonate’s mother, real-time noncontact visit program, COVID-19, nurses’ support, stress, NICU

## Abstract

Background: With the spread of COVID-19, neonatal intensive care units restricted visiting hours to prevent infection. As a result, mothers of high-risk newborns were restricted from making contact with their children. Various problems could be encountered for hospitalized children and mothers of high-risk newborns due to restrictions on visits in the neonatal intensive care unit. Therefore, during the pandemic, continuous nursing support for mothers of high-risk newborns was needed. Methods: In this study, a nonequivalent control group non-synchronized design was employed. The subjects of the study were 36 mothers of high-risk neonates (20 in the experimental group and 16 in the control group) in E University Hospital, D Metropolitan City, from July to September 2022. The experimental group received a 10 min program performed by a nurse from 7:00 p.m. to 9:00 p.m. three times per week. Results: There were significant differences in nurses’ support between the experimental and control groups (F = 25.594, *p* < 0.001), changes over time (F = 16.178, *p* < 0.001), and time–group interactions (F = 9.663, *p* = 0.003). Conclusions: It was interpreted that the real-time noncontact visitation program could transcend time and space for many mothers of high-risk newborn babies, who suffered psychologically due to restrictions and bans on visitations during the COVID-19 pandemic.

## 1. Introduction

With the global spread of COVID-19, which was first identified in Wuhan, China, in 2020, the World Health Organization (WHO) eventually announced the pandemic on 11 March 2020 [1]. South Korea failed to avoid the impact of the global spread of COVID-19 [2]. With the spread of COVID-19, hospitals delivered coping guidelines in each period to minimize any contact with the outside. For hospital visits, self-restriction, restriction, and prohibition stages were announced, followed by prohibition extending to noncontact visits [2]. These principles of infection prevention by hospitals led to restrictions on visits, specifically on families’ direct/indirect participation in caregiving or decision making [3].

Before the pandemic, emphasis was placed on the importance of direct interactions with the mothers of high-risk neonates, parent participation, and rapport with medical staff during hospitalization from the perspective of family based care [4,5]. However, the current restrictions on visits to neonatal intensive care units (NICUs) can raise diverse issues among the high-risk neonates in the units and their mothers. For this reason, nurses need to provide continuous support to the mothers of high-risk neonates during their stay in NICUs [6].

While the successful birth of high-risk neonates is highly valued and provides parents with happiness, often representing a moment in their life that they will never forget, it can also be a stressful event. A high-risk neonate’s postnatal stay in an NICU can make their mother’s experience more stressful [7].

The neonates principally entering NICUs include premature babies, underweight infants, and those with perinatal asphyxia, respiratory disorders, heart diseases, infection, congenital malformation, jaundice, and inborn errors of metabolism. They require a stay in the NICU and interventional treatment [8].

NICUs have restrictions on visiting times to prevent infection; for this reason, high-risk neonates’ mothers have restricted contact with their children. High-risk neonates’ mothers are more likely to experience depression, guilt, sadness, anger, and helplessness than those of normal infants, due to the healthcare environment of NICUs, their children’s unstable conditions, uncertainty regarding their children’s conditions and prognosis, and the loss of maternal roles that they expected to have following giving birth to a healthy child [9,10].

High-risk neonates’ mothers experience severe emotional stress due to the visible course of treatment of their children’s conditions and anxiety about convalescence. The replacement of mothers with nurses for high-risk neonatal childcare makes mothers confused about normal maternal roles. In addition, a poor rapport with medical staff and the burden of medical costs make high-risk neonates’ mothers experience more stress [11,12]. Not only can the stress cause negative relationships and family maladjustment that interfere with the interactions between high-risk neonates and their mothers, but it can also contribute to parenting stress and a lack of self-confidence in parenting [13].

During this period, nurses need to identify specific types of stress so that high-risk neonates’ mothers can adapt to the fear caused by the transformed maternal roles and the resulting stress, and nurses need to develop and provide supportive nursing interventions for them [14]. Nurses’ support reduces postnatal depression among high-risk neonates’ mothers [15]; makes them less likely to experience anxiety, depression, and stress [16]; and helps them to form attachments with their children and to make family adjustments through emotional adjustments [17]. 

High-risk neonates’ mothers consider nurses to be helpful and reliable [18]. As the primary caregivers who are most frequently in contact with neonates, nurses are not only required to understand and intervene in the stress experienced by high-risk neonates’ mothers, but also to provide effective support so that high-risk neonates’ mothers can properly adapt themselves to the healthcare environment of NICUs and their transformed maternal roles [19]. 

In this context, consideration has been given to the need for noncontact visiting services, and hospitals have positively reviewed the introduction of relevant service plans [20].

### Purpose

The purpose of this study was to prevent infection caused by COVID-19.

The effectiveness was verified by developing and applying a real-time contactless visit program for high-risk newborn mothers who entered the neonatal intensive care unit.

Specific purposes included the following:

(1) Identify the impact of a real-time noncontact visit program on nurses’ support.
(2) Identify the impact of a real-time noncontact visit program on the stress of mothers.

## 2. Materials and Methods

### 2.1. Research Design

In this study, a nonequivalent control group non-synchronized design was used to develop a real-time noncontact visit program for the mothers of high-risk neonates in NICUs with restricted visits due to COVID-19, and to verify the effects of this program.

### 2.2. Subjects

**Figure 1:**
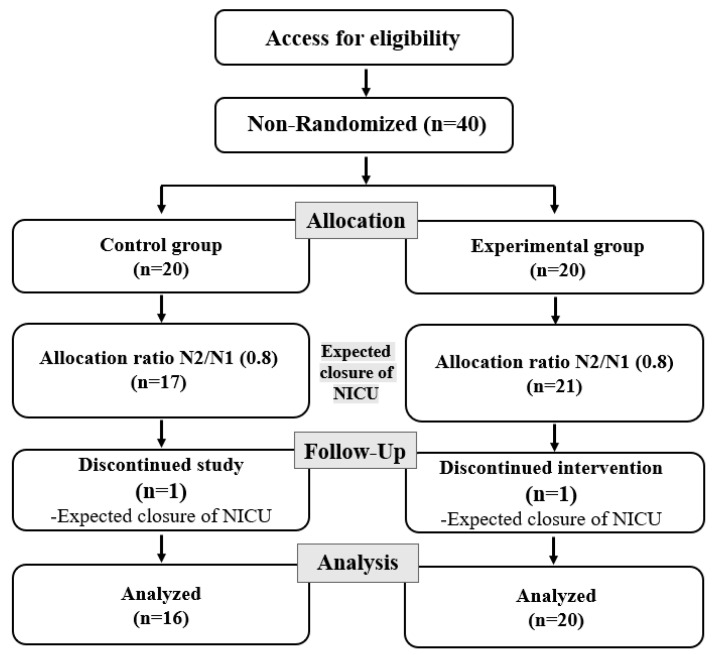
Process flow diagram.

The sample size was estimated using the G*Power 3.1.9.4 program. With an effect size of d = 0.95, a significance level of α = 0.05, and a testability of 1 − β = 0.8, which were based on Cohen’s sample size estimation table and existing research [21], the estimated sample size was 38 (19 in each group), and the selected size was 40, taking a dropout rate of 5% into account. To prevent the spread and contamination of experimental effects, data collection was not synchronized between the control group and the experimental group. However, the policy for the interruption of NICU operation at E University Hospital in D Metropolitan City during data collection from the control group forecasted difficulty in conducting the research. Taking the NICU closure timeline into account, the allocation ratio of N2/N1 0.8 was inevitably applied to re-estimate the control group at 17 and the experimental group at 21. However, the closure of the neonatal intensive care unit was earlier than expected, so one in the control group and one in the experimental group were excluded from the number of subjects, as shown in Figure 1.

The inclusion criteria for the subjects were as follows:

- High-risk newborns’ mothers who understood the purpose of this study and agreed to participate in it;
- High-risk newborns’ mothers who understood the contents of the questionnaire and completed it;
- High-risk newborns’ mothers whose children had never stayed in NICUs.

The exclusion criteria for the subjects were as follows:

- Mothers of high-risk newborns who stayed in NICUs for less than 48 h;
- Mothers of re-hospitalized high-risk newborns.

High-risk newborns: Premature (less than 2.5 kg or less than 37 weeks), intrauterine growth restriction (IUGR), large for gestational age (LGA), transient tachypnoea of newborn (TTN), idiopathic respiratory distress syndrome (IRDS), meconium aspiration syndrome, seizure, hypoglycemia, asphyxia, etc.

### 2.3. Instruments

#### 2.3.1. NPST: Nurse–Parent Support Tool

The Nurse–Parent Support Tool (NPST), which was developed by Miles, Carlson, and Brunssen (1999) [22] and translated into Korean by Han and Chae (2016) [23], was used to measure nurses’ support as perceived by the high-risk neonates’ mothers. This tool is composed of 21 items in 4 areas: communicative and informative support provided to high-risk neonates’ mothers, support that respects the mothers’ roles, emotional support, and caring support provided to the children. It uses a five-point Likert scale, with the scores ranging from one (totally negative) to five (totally positive). A higher score indicates a higher level of nurses’ support. For reliability, Cronbach’s α was 0.94, in Han and Chae (2016) [23]. For reliability of the internal consistency in this study, Cronbach’s α was 0.91.

#### 2.3.2. PSS: Parental Stressor Scale

A tool developed by Miles et al. (1992) [24], which was adapted by Miles et al. (1993) [25] and translated into Korean by Kim (2000) [26], was used to measure the stress experienced by the mothers of the high-risk neonates. The Parental Stressor Scale (PSS): Neonatal Intensive Care Unit was used to measure the perception of stress caused by the physical and psychosocial environment of NICUs among the high-risk neonates’ mothers. This scale is composed of 26 items in such areas as the environment of NICUs (5); children’s appearance, behavior, and treatment (14); and the relationships between high-risk neonates and their mothers and changes in maternal roles (7). This uses a five-point Likert scale, with the scores ranging from one (never feel) to five (very strongly feel); a higher score indicates a higher level of stress experienced by the high-risk neonates’ mothers. With reference to the tool developed by Miles et al. (2007) [27], any item not applicable to neonates was indicated as being not applicable and scored zero. For reliability, Cronbach’s α was estimated at 0.94 in Miles et al. (1993) [25] and at 0.93 in Kim (2000) [26] at the time of its development. For the reliability of internal consistency in this study, Cronbach’s α was 0.93.

### 2.4. Program Development

To conduct this study, the researcher, one professor of pediatric nursing, three registered nurses with at least ten years of career experience in NICUs, and two professional nurses developed a real-time noncontact visit program in the NICU of E University Hospital in D Metropolitan City. The NICU real-time noncontact visit program for COVID-19 prevention was developed on the basis of the analysis, design, development, implementation, and evaluation (ADDIE) model.

At the analysis stage, a literature review regarding nurses’ support as perceived by the high-risk neonates’ mothers, stress level, and the quality of healthcare was performed in terms of the prohibition of face-to-face visits in the COVID-19 pandemic situation. The amount of time using a smartphone and the reason for its use, characteristics, and current status were investigated to determine the degree of demand for visits using smartphone-based video calls in the case of face-to-face visit prohibition. 

At the design stage, the contents of the real-time noncontact visit program were constructed, sequenced, and allocated time.

The introduction part began with an accurate identification, which involved a nurse’s self-introduction and greeting to a high-risk neonate’s mother. Next, the general appearance of the child and the surroundings of the NICU were shown to the mother. The development part provided a description of the high-risk neonate’s basic vital signs and the details of the nursing activities provided (V/S, body weight, diet, umbilical cord management, infection management, skin status, ventilator application, respiration status, etc.). The mother was also provided with educational information regarding “how to manage and cope with a sick child” and motivated to play maternal roles through Q&A. The teaching contents were categorized into a total of four areas: bathing, diaper changing and diaper rash control, milk powder formulations and breast milk preparation, and car safety. In the conclusion part, the mother was asked to deliver a message with facial expressions and her voice through the screen.

On the basis of the functions and components of the video call, which were defined at the design stage, the program was named a “real-time noncontact visit program” at the development stage. It was designed to send five pieces of teaching materials converted into PDF files via SMS or Kakao so that the subjects could be provided with a chance for visual immersion, repetitive acquisition, and memorization.

At the implementation stage, each of the two nurses with at least ten years of career experience in NICUs were asked to carry out a pilot application to two mothers of high-risk neonates according to the program’s procedure and schedule.

At the evaluation stage, the problems and errors of the program were investigated among both the mothers and the nurses who were involved in the pilot application of the program. During the development of the real-time noncontact visit program, the problems and improvements were constantly discussed and revised.

As a mediator on the video call, the nurse was asked to show the neonate. The nurse stayed silent so that the high-risk neonate and the mother could interact with each other. The mother was asked to deliver a desired message (vocal or video materials, including songs, fairy tales, and poems) to her child through the screen. At the end of the ten-minute intervention with the real-time noncontact visit program, the video call ended; then, the nurse sent five pieces of teaching materials converted into PDF files to the mother via SMS or Kakao. The program composition is shown in Table 1.

### 2.5. Research Procedure

This study was conducted from July to September 2022 after obtaining approval from the Institutional Review Board (IRB) of E University Hospital in D Metropolitan City. The chief of the NICU, the nursing staff, and the chief of the ward were provided with an explanation of the purpose and methods of the study to obtain permission before starting the study. The high-risk neonates’ mothers who consented to participate in the study met the eligibility criteria. 

**Figure 2:**
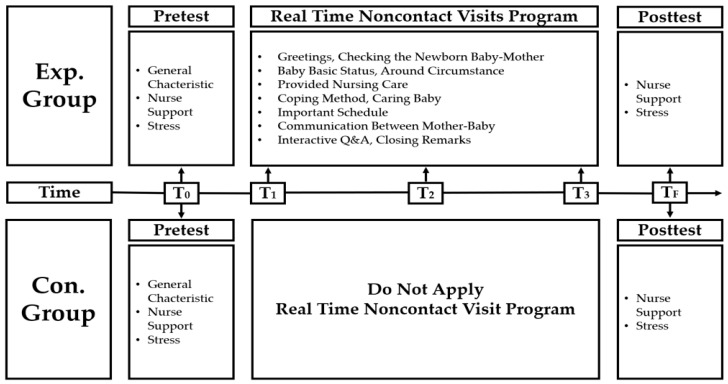
Data collection procedure flow diagram.

The control group first participated in the study in order to prevent the spread of experimental effects. The control group was formed, followed by the experimental group, in each period according to the order of hospitalization. All the subjects consenting to participation took a pretest comprising a questionnaire regarding general characteristics, the nurses’ support as perceived by the high-risk neonates’ mothers, and the stress that they experienced. Immediately after the pretest, the real-time noncontact visit program was applied to the experimental group consenting to participate in the study. Registered nurses on three shifts in the NICU implemented the program from 7 to 9 o’clock during the evening shift, three times a week (Tuesday, Thursday, and Saturday). It took 10 min to implement the real-time noncontact visit program. Seven days after hospitalization for the control group and after the application of the intervention in three sessions with the real-time noncontact visit program for the experimental group, a posttest was performed using the same questionnaire as in the pretest, regarding the nurses’ support as perceived by the high-risk neonates’ mothers and the stress that they experienced. The data collection procedure is shown in Table 2 and Figure 2.

### 2.6. Data Analysis

The collected data were analyzed using SPSS/WIN 26.0. The real number, percentage, and mean and standard deviation were used to analyze the subjects’ general and principal dependent variables. The pretest for homogeneity was performed using the χ^2^ test, *t*-test, and ANOVA on the basis of the general characteristics and primary dependent variables for both groups. The effects of the program were analyzed using a *t*-test. 

### 2.7. Ethical Considerations

This study was approved by the IRB of E University Hospital in D Metropolitan City (EMC 2022-05-002-003). The chief of the NICU, the nursing staff, and the chief of the ward were provided with an explanation of the purpose and methods of the study to obtain permission before starting the study. For confidentiality reasons, the questionnaires were sealed immediately after distribution and completion. The subjects were provided with an explanation stating that they could discontinue completing the questionnaire during the study and that the questionnaire would not be used for any purpose other than the study, and they were asked to provide written consent to participation in the study, which contained the explanation before starting the survey.

## 3. Results

### 3.1. Homogeneity Test of General Characteristics of High-Risk Neonates’ Mothers

The homogeneity test of the general characteristics of the high-risk neonates’ mothers confirmed between-group homogeneity, with no significant difference in age, marital status, occupation, education, religion, family type, family size, birth order, planned pregnancy, the perception of the child’s condition and prognosis, the target of reliance, or the degree of reliance on the medical staff (Table 3).

### 3.2. Homogeneity Test of General Characteristics of High-Risk Neonates

The homogeneity test of the general characteristics of the high-risk neonates confirmed between-group homogeneity, with no significant difference in the child’s sex, birth weight, diagnosis, surgery plan, surgery implementation, ventilator application, use of an incubator, feeding method, or feeding type (Table 4).

### 3.3. Pretest of Homogeneity in Participants’ Dependent Variables

Regarding prior nurse support as perceived by the high-risk neonates’ mothers, the experimental group scored 65.60 ± 13.44 and the control group scored 59.06 ± 13.03. Regarding prior stress experienced by the high-risk neonates’ mothers, the experimental group scored 108.4 ± 13.59 and the control group scored 103.75 ± 17.93. The pretest of homogeneity in the dependent variables confirmed prior between-group homogeneity, with no statistically significant difference in nurse support perceived by the high-risk neonates’ mothers or the stress that they experienced (Table 5).

### 3.4. Comparison of Nurses’ Support of High-Risk Newborns’ Mothers

**Figure 3:**
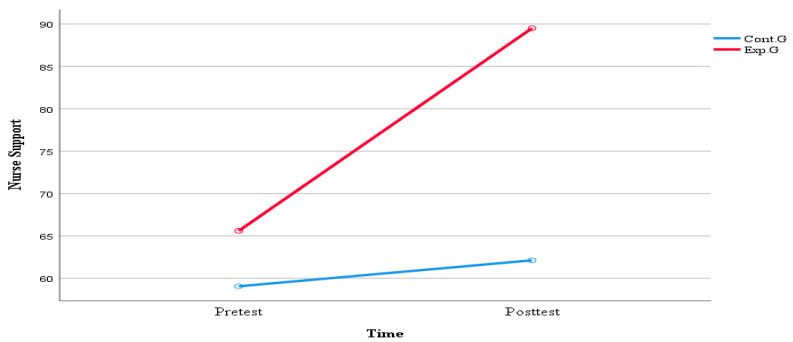
Comparison of nurses’ support of high-risk newborns’ mothers between the two groups.

Table 6 and Figure 3 show the comparison of nurses’ support in high-risk neonatal mothers based on the application of the real-time contactless visit program. Changes in nurses’ support before and after the program were analyzed using ANOVA. There were significant differences between the two groups (F = 25.594, *p* < 0.001), changes over time (F = 16.178, *p* < 0.001), and time–group interactions (F = 9.663, *p* = 0.003) in nurses’ support.

### 3.5. Comparison of Stress of High-Risk Newborns’ Mothers 

**Figure 4:**
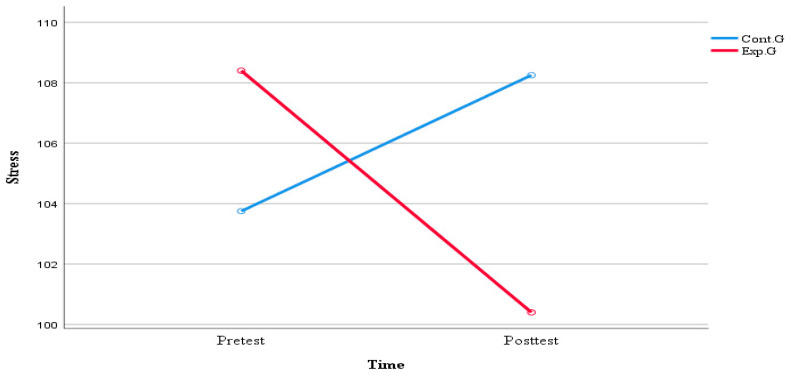
Comparison of stress of high-risk newborns’ mothers between the two groups.

Table 7 and Figure 4 show the comparison of stress in high-risk neonatal mothers based on the application of the real-time contactless visit program. Changes in stress before and after the program were analyzed by ANOVA. There was no significant difference between the two groups (F = 0.112, *p* = 0.739). In addition, there were no significant differences in changes over time (F = 0.134, *p* = 0.716) and time–group interactions (F = 1.703, *p* = 0.195).

## 4. Discussion

This study was conducted to develop and apply a real-time noncontact visit program for high-risk neonates’ mothers and to test its effects on nurses’ support, as perceived by the mothers, and on the stress experienced by the mothers. While all visits to NICUs were limited due to the COVID-19 pandemic, the high-risk neonates’ mothers in the experimental group were provided with the real-time noncontact visit program in three sessions, and those in the control group kept using the existing type of visit. 

The real-time noncontact visit program was developed on the basis of the ADDIE model. Most of the previous studies aimed at developing a program targeting high-risk neonates [28,29] have an analysis stage composed of a literature review and interviews. Research on the development of educational programs [30] has indicated that an analysis of learners’ needs is a very important element in setting the goal of the program and in designing a rational and valid program based on the learners’ needs. Similarly, research on the needs for smartphone-based education in the noncontact phase, with restricted visits in pursuit of infection prevention [31], has shown that the parents of preschoolers had a higher demand for smartphone-using education than those of children in other age groups.

The real-time noncontact visit program was designed for high-risk neonates and their mothers to create time to develop mother–infant attachment. High-risk neonates’ mothers are mentally and physically closest to their children and play a crucial role in their children’s healthy growth and development. High-risk neonates and their mothers get to know each other through constant interactions, undergo a process of dynamic regulation, and become attached to each other accordingly [32]. On this basis, this study helped them to promote healthy interactions by ensuring that the mothers and babies showed the maximum reaction to each other, even if they were unable to make direct contact or get close enough to form substantial bonds due to the various restrictions.

This study’s aim of developing and applying a real-time noncontact visit program is somewhat similar to research aimed at developing noncontact visiting applications [33], in that both types of research extend consideration to real-time noncontact visiting services, as the need for visits was recognized at the time of restricted hospital visits in the COVID-19 pandemic era. However, this study is significant in that it constitutes experimental research with the aims of applying a real-time noncontact visit program and testing its effects on nurses’ support as perceived by high-risk neonates’ mothers, as well as on the mothers’ stress.

This study confirmed that nurses’ support as perceived by the experimental group provided with the real-time noncontact visit program increased, contrary to the control group, which kept using the existing type of visit. The literature review [28,34] revealed that, during high-risk neonates’ hospitalization, nurses were identified as the medical persons closest to the mothers and provided and supported the nursing that they needed. 

High-risk neonates’ mothers expect to obtain knowledge about caring for neonates, about their children’s medical status, and informative support relevant to maternal roles [35]. Informative support has been found to have positive effects on self-confidence in carrying out maternal roles among high-risk neonates’ mothers [36]. High-risk neonates’ mothers may experience stress from anxiety and the fear of unpredictable treatment [12], and they may constantly ask questions due to a lack of information about their children’s prognosis, treatment, and procedure. Nurses are in a position to help high-risk neonates’ mothers most rapidly and can provide them with positive support. For this reason, nurses need to study effective information delivery intervention methods and make careful considerations. 

The aim of this study was to teach mothers how to treat and cope with sick children via a real-time noncontact visit program and to provide them with informative support. The real-time noncontact visit program confirmed the possibility of “noncontact visits” and provides basic data from which to develop an education program. Taking into consideration the findings of the literature review, that providing both emotional and informative support is more effective in relieving stress and anxiety than providing either one separately [37], it is necessary to complement the real-time noncontact visit program with nurses’ support at multilateral levels. 

It is suggested that further research be conducted to test various methods of teaching interventions so that the informative support can be positive. For example, it is possible to compare the effects of informative support among vivid real-time teaching interventions, such as video calls and non-real-time teaching materials, for example, graphic files providing repetitive access to teaching materials or PDF files, and many other intervention methods. 

On the basis of the differences between the experimental group that was provided with the program and the control group, it was found that the real-time noncontact visit program contributed to the nurses’ support as perceived by the high-risk neonates’ mothers and that there was no difference in the levels of stress. This result is consistent with the findings of the literature review that factors such as the child’s conditions and prognosis, and the hospital environment, are significant stressors [12]. In particular, mothers experienced the hardship of being unable to hold their babies, undergoing a long duration of treatment for their babies, and seeing their babies in distress. When a high-risk neonate enters a NICU, the mother may experience high stress due to helplessness because she is unable to protect her child from distress or pain [38]. During hospitalization, concerns about the child’s health status, errors in communication with the medical staff, and a lack of information increase stress [39]. The more severe the high-risk neonate’s condition and the more concerned the mother is about her child, the more negative emotions, such as anxiety and depression, that the mother experiences [40]. High-risk neonates’ mothers who consider their children to be unhealthy are more likely to have postnatal depression [29]. On this basis, postnatal depression must also have a considerable impact on the stress experienced by the high-risk neonates’ mothers. In the COVID-19 pandemic situation with restricted NICU visits, high-risk neonates’ mothers are expected to experience more stress because they have almost no chance of participating in childcare, and nurses have a lack of time to provide them with their full support.

Because the real-time noncontact visit program in this study is implemented via smartphone-based video calls, and high-risk neonates’ mothers are not able to see their children personally before their eyes—only on the screen—there can be errors in their recognition of their children’s conditions. For this reason, high-risk neonates’ mothers are more likely to become depressed and anxious. When their children become better, are discharged from hospital, and become a member of the family, the mothers may develop maternal roles through interactions during the course of the relationship with their children, and their stress may be relieved. Therefore, nurses need to make efforts to identify the basic causes of the stress experienced by high-risk neonates’ mothers and to relieve their stress through encouragement and support.

The limitations of this study are as follows: (1) The subjects of this study were limited to mothers of high-risk newborns admitted to the neonatal intensive care unit of a university hospital located in D Metropolitan City. Considering the severity of the patients’ conditions and the size of the hospital, there are limitations in generalizing the results to all mothers of high-risk newborns; (2) Due to the lack of previous studies measuring the support and stress of nurses perceived by mothers of high-risk newborns in relation to special situations such as the COVID-19 pandemic and noncontact visits in the neonatal intensive care unit, it is difficult to compare the results of this study; (3) This study was conducted in the context of COVID-19, so there are limitations in interpreting the results, considering the difficulties caused by the continuous changes in visitation procedures and unexpected circumstances, such as the closure of the neonatal intensive care unit; (4) In this study, there were limitations in interpreting the results because the categories of health status and the hospitalization period of high-risk newborns, which change frequently, were different for each patient.

The strengths of this study are as follows: It was verified that the support of nurses perceived by mothers of high-risk newborns who received the real-time noncontact visitation program was improved. This is expected to be an essential intervention in the Neonatal Intensive Care Unit, where partnerships with medical staff are important, by enabling smooth rapport between mothers and nurses of high-risk newborns even in the face of limited visits due to COVID-19. This will ultimately increase the satisfaction with medical services.

## 5. Conclusions

These results demonstrate that the real-time noncontact visit program failed to relieve the stress that the high-risk neonates’ mothers experienced but was effective in increasing nurses’ support as perceived by the mothers. The real-time noncontact visit program supported by these results is expected to increase nurses’ support as perceived by the mothers of high-risk neonates in NICUs and to be useful as an intervention for controlling stress.

The real-time noncontact visit program in this study can permit visits, without temporal or spatial restrictions, to a large number of high-risk neonates’ mothers experiencing mental distress due to restricted and prohibited visits in the COVID-19 pandemic situation. Even in noncontact situations, it enables communication between the medical staff and the high-risk neonates’ mothers, and it encourages participation in the course of treatment. Forming a positive rapport between high-risk neonates’ mothers and medical staff will be an intervention that is essential to the decision making regarding the treatment for high-risk children in NICUs. In addition, this visit program is expected to be applicable to many other situations: the wards that immunocompromised patients stay in, adult ICUs, and high-risk neonates’ mothers residing in other regions or overseas.

## Figures and Tables

**Table 1:** Real-time noncontact visit program contents.

1. Introduction (1 min)	
Greetings and identifying newborns and mothers	
2. Development (7 min)	
(1) Baby basic status, circumstances	Vital signs
	Weight
	Feeding volume, how to eat, and what to eat
(2) Provided nursing care	Taking care of newborn’s eyes and umbilical cord
	Bathing and skincare
	Maintaining airway, breathing, and body temperature of the newborn
	Breast feeding or powdered milk feeding
	Fall prevention
	Infection control
	Congenital metabolic abnormality test
	Hearing test
(3) Coping method, caring for baby	How to bathe the baby (Day 1)
	How to change diapers (Day 2)
	Diaper rash prevention and management (Day 2)
	Preparation of infant formula (Day 3)
	Car safety (Day 3)
(4) Important schedule	Note the date of the interview with the professor
	Discharge scheduled
(5) Communication between mother and baby	Delivering a message that a mother wants to convey to her child (voice, video, song, fairy tale, etc.)
3. Conclusion (two minutes)	
Interactive Q&A, closing remarks	

**Table 2:** Real-time noncontact visit program flow.

Time	Stage	Group		Procedure
T_0_	Pretest	Con. Group	1	When explaining the hospitalization notice in the education room on the day of admission to the neonatal intensive care unit, this study was described, and the purpose and contents were explained.
			2	Subjects who agreed to participate in the study responded to questions regarding 22 general characteristics, 21 nurse support questions, and 26 stress questions. The questionnaire response time was about 15 min.
T_4_	Posttest	Con. Group	1	After three interventions in the real-time noncontact visit program, mothers of high-risk newborns responded to the same questionnaire as the preliminary survey.
			2	The subjects responded to 22 questions regarding general characteristics, 21 questions regarding nurse support, and 26 questions regarding stress, which were the same as in the preliminary survey. The questionnaire response time was about 15 min.
			3	An e-gift card (for example) was provided to the subject who completed the post-investigation.
T_0_	Pretest	Exp. Group	1	When explaining the hospitalization notice in the education room on the day of admission to the neonatal intensive care unit, this study was described, and the purpose and contents were explained.
			2	Subjects who agreed to participate in the study responded to questions regarding 22 general characteristics, 21 nurse support questions, and 26 stress questions. The questionnaire response time was about 15 min.
T_1_~T_3_	Apply treatment Real-time noncontact visit program	Exp. Group	1	A real-time noncontact visit program was applied using a smartphone in the neonatal intensive care unit with the contact information provided by the subject.
			2	The real-time noncontact visit program was conducted between 7 p.m. and 9 p.m. three times a week (Tuesday, Thursday, and Saturday) by nurses with over 10 years of work experience in the neonatal intensive care unit. The time required for the developed visit program was 10 min. At the beginning of the real-time noncontact visit program intervention, the subject was familiarized with the unfamiliar environment by showing them the overall appearance and surrounding environment on the screen, starting with a brief introduction and greeting by the nurse. The basic vital signs of high-risk newborns and the nursing activities provided (V/S, weight, diet, discharge management, infection control, skin condition, ventilator application, respirator application, respiratory health, etc.) were explained. Educational information was also delivered, relating to how to handle and cope with patients, and motivated the role of mothers through simple questions and answers. The contents of the education program were classified into four categories: bathing, diaper replacement and diaper rash management, formula preparation and breast milk preparation, and caution when riding in a car.
			3	Through a video call, a nurse, who was an intermediary, was asked to show the newborn. The nurse remained silent so that the mother and high-risk newborn could communicate. High-risk newborn mothers were asked to deliver messages (voice or video materials such as songs, fairy tales, poems, etc.) to their children through the screen. After the 10-min real-time noncontact visit program intervention, the nurse sent five educational materials converted into PDF files to the mother of the high-risk newborn through SMS or Kakao.
T_4_	Posttest	Exp. Group	1	After three interventions in the real-time noncontact visit program, mothers of high-risk newborns responded to the same questionnaire as the preliminary survey.
			2	The subjects responded to 22 questions regarding general characteristics, 21 questions regarding nurse support, and 26 questions regarding stress, which were the same as in the preliminary survey. The questionnaire response time was about 15 min.
			3	An e-gift card was provided as an example to the subject who completed the post-investigation.

**Table 3:** Homogeneity test of general characteristics of high-risk newborns’ mothers.

Variables	Categories	Exp. (n = 20)	Cont. (n = 16)	χ^2^	*p*
		N (%)	N (%)		
Age (years)	20~29	3 (15.0%)	0 (0.0%)	15.027	0.306
	30~39	16 (80.0%)	13 (81.9%)		
	≥40	1 (5.0%)	3 (18.8%)		
Employment	Yes	8 (40.0%)	10 (62.5%)	1.800	0.180
	No	12 (60.0%)	6 (37.5%)		
Education	Middle school	1 (5.0%)	0 (0.0%)	1.957	0.581
	High school	6 (30.0%)	3 (18.8%)		
	College	11 (55.0%)	12 (75.0%)		
	Graduate school	2 (10.0%)	1 (6.3%)		
Religion	Have no religion	9 (45.0%)	10 (62.5%)	1.647	0.649
	Christianity	7 (35.0%)	4 (25.0%)		
	Catholicism	1 (5.0%)	0 (0.0%)		
	Buddhism	3 (15.0%)	2 (12.5%)		
Monthly income	<200	2 (10.0%)	0 (0.0%)	0.347	0.261
	200~400	10 (50.0%)	6 (37.5%)		
	>400	8 (40.0%)	10 (62.5%)		
Family form	Nuclear family	19 (95.0%)	15 (93.8%)	0.026	0.871
	Large family	1 (5.0%)	1 (6.3%)		
Number of family members	3	12 (60%)	6 (37.5%)	4.690	0.321
	4	6 (30.0%)	7 (43.8%)		
	5	0 (0.0%)	2 (12.5%)		
	6	1 (5.0%)	0 (0.0%)		
	7	1 (5.0%)	1 (6.3%)		
Birth order	1	12 (60.0%)	6 (37.5%)	3.600	0.308
	2	7 (35.0%)	7 (43.8%)		
	3	0 (0.0%)	2 (12.5%)		
	4	1 (5.0%)	1 (6.3%)		
Planned pregnancy	Yes	16 (80.0%)	13 (81.3%)	0.009	0.925
	No	4 (20.0%)	3 (18.8%)		
Recognition of the improvement in the child’s condition	Know	9 (45.0%)	9 (56.3%)	2.665	0.446
	Know A little bit	7 (35.0%)	6 (37.5%)		
	Know very little	1 (5.0%)	1 (6.3%)		
	Have no idea	3 (15.0%)	0 (0.0%)		
The most helpful person	Nurse	8 (40.0%)	5 (31.3%)	1.466	0.690
	Doctor	1 (5.0%)	1 (6.3%)		
	Husband	11 (55.0%)	9 (56.3%)		
	Real mother	0 (0.0%)	1 (6.3%)		
Medical staff trust	Trust	5 (25.0%)	4 (25.0%)	0.675	0.714
	More trust	9 (45.0%)	9 (56.3%)		
	Very trusting	6 (30.0%)	3 (18.8%)		

Exp. = experimental group; Cont. = control group.

**Table 4:** Homogeneity test of general characteristics of high-risk newborns.

Variables	Categories	Exp. (n = 20)	Cont. (n = 16)	χ^2^	*p*
		N (%)	N (%)		
Number of days in hospital	≤10	2 (10.0%)	4 (25.2%)	27.225	0.398
	11–20	6 (30.0%)	6 (37.7%)		
	21–30	11 (55.0%)	1 (6.3%)		
	31–40	0 (0.0%)	1 (6.3%)		
	≥41	1 (5.0%)	4 (25.2%)		
Sex	Boy	13 (65.0%)	8 (50.0%)	0.823	0.364
	Girl	7 (35.0%)	8 (50.0%)		
Weight	<2 kg	1 (5.0%)	4 (25.2%)	36	0.422
	2 kg–3 kg	10 (50.0%)	6 (37.8%)		
	3.01 kg–4 kg	7 (35.0%)	5 (31.5%)		
	≥4.01 kg	2 (10.0%)	1 (6.3%)		
Diagnostic name	Premature	7 (35.0%)	7 (43.8%)	5.625	0.229
	Meconium stain	1 (5.0%)	0 (0.0%)		
	RDS	12 (60.0%)	6 (37.5%)		
	Jaundice	0 (0.0%)	1 (6.3%)		
	Sepsis	0 (0.0%)	2 (12.5%)		
Ventilator	Yes	11 (55.0%)	4 (25.0%)	3.291	0.070
	No	9 (45.0%)	12 (75.0%)		
Feeding method	NPO	5 (25.0%)	3 (18.8%)	0.776	0.855
	Gastrointestinal	9 (45.0%)	6 (37.5%)		
	Gastrointestinal + oral	2 (10.0%)	2 (12.5%)		
	Oral	4 (20.0%)	5 (31.3%)		
Feeding type	Normal formula	9 (45.0%)	2 (12.5%)	6.070	0.108
	Special formula	2 (10.0%)	4 (25.0%)		
	Breastfeeding	4 (20.0%)	7 (43.8%)		
	NPO	5 (25.0%)	3 (18.8%)		

Exp. = experimental group; Cont. = control group.

**Table 5:** Homogeneity test of dependent variables between the two groups.

Category	Exp. (n = 20)	Cont. (n = 16)	*t*	*p*
	M ± SD	M ± SD		
Nurse support	65.60 ± 13.44	59.06 ± 13.03	1.475	0.150
Stress	108.4 ± 13.59	103.75 ± 17.93	0.859	0.398

Exp. = experimental group; Cont. = control group.

**Table 6:** Comparison of nurses’ support of high-risk newborns’ mothers between the two groups.

Variable	Time	Exp. (n = 20)	Cont. (n = 16)	Source	F	*p*
		M + SD	M + SD			
Nurses’ support	Pretest	65.60 ± 13.44	59.06 ± 13.03	Group	25.594	<0.001
	Posttest	89.50 ± 15.03	62.13 ± 14.85	Time	16.178	<0.001
				G × T	9.663	0.003

Exp.= Experimental group; Cont. = Control group.

**Table 7:** Comparison of stress of high-risk newborns’ mothers between the two groups.

Variable	Time	Exp. (n = 20)	Cont. (n = 16)	Source	F	*p*
		M + SD	M + SD			
Stress	Pretest	108.40 ± 13.59	103.80 ± 17.93	Group	0.112	0.739
	Posttest	100.40 ± 29.71	108.30 ± 13.23	Time	0.134	0.716
				G × T	1.703	0.196

Exp. = Experimental group; Cont. = Control group.

## Data Availability

The data that support the findings of this study are available from the corresponding author upon reasonable request.
